# Value of original and modified pathological scoring systems for prognostic prediction in paraffin-embedded donor kidney core biopsy

**DOI:** 10.1080/0886022X.2024.2314630

**Published:** 2024-02-12

**Authors:** Jinghong Tan, Huanxi Zhang, Longshan Liu, Jun Li, Qian Fu, Yan Li, Chenglin Wu, Ronghai Deng, Jiali Wang, Bowen Xu, Wenfang Chen, Shicong Yang, Changxi Wang

**Affiliations:** a Organ Transplant Center, The First Affiliated Hospital, Sun Yat-sen University, Guangzhou, China; b Guangdong Provincial Key Laboratory on Organ Donation and Transplant Immunology, Guangzhou, China; c Guangdong Provincial International Cooperation Base of Science and Technology (Organ Transplantation), Guangzhou, China; d Department of Pathology, The First Affiliated Hospital, Sun Yat-sen University, Guangzhou, China; e Department of Nephrology, The First Affiliated Hospital, Sun Yat-sen University, Guangzhou, China

**Keywords:** Kidney transplantation, pretransplant donor kidney biopsy, core biopsy, pathology, glomerulosclerosis, eGFR

## Abstract

**Background:**

No study has validated, compared and adapted scoring systems for prognosis prediction based on donor kidney core biopsy (CB), with less glomeruli than wedge biopsy.

**Methods:**

A total of 185 donor kidney CB specimens were reviewed using seven scoring systems. The association between the total score, item scores, score-based grading, and allograft prognosis was investigated. In specimens with less than ten glomeruli (88/185, 47.6%), scoring systems were modified by adjusting weights of the item scores.

**Results:**

The Maryland aggregate pathology index (MAPI) score-based grading and periglomerular fibrosis (PGF) associated with delayed graft function (DGF) (Grade: OR = 1.59, *p* < 0.001; PGF: OR = 1.06, *p* = 0.006). Total score, score-based grading and chronic lesion score in scoring systems associated with one-year and 3-year eGFR after transplantation. Total-score-based models had similar predictive capacities for eGFR in all scoring systems, except MAPI and Ugarte. Score of glomerulosclerosis (GS), interstitial fibrosis (IF), tubular atrophy (TA), and arteriolar hyalinosis (AH) had good eGFR predictive capacities. In specimens with less than ten glomeruli, modified scoring systems had better eGFR predictive capacities than original scoring systems.

**Conclusions:**

Scoring systems could predict allograft prognosis in paraffin-embedded CB with ten more glomeruli. A simple and pragmatic scoring system should include GS, IF, TA and AH, with weights assigned based on predictive capacity for prognosis. Replacing GS scores with tubulointerstitial scores could significantly improve the predictive capacity of eGFR. The conclusion should be further validated in frozen section.

## Introduction

In patients with end-stage renal disease (ESRD), kidney transplantation is the best treatment. However, the demand for kidney transplantation exceeds the availability of standard criteria donors (SCD), leading to an increase in expanded criteria donors (ECD). Pre-transplant donor kidney biopsy (PTDB) has been widely employed in assessing donor kidney. It assists clinicians in determining whether to accept or discard donor kidneys. Chronic lesions, including glomerulosclerosis (GS), tubular atrophy (TA), interstitial fibrosis (IF), and pathological vascular changes, are emphasized in PTDB scoring systems.

Over the last two decades, multiple histopathological scoring systems [1–7] have been developed based on PTDB. These systems demonstrate differences (Table S1). First, Banff donor scoring system incorporates acute lesions like glomerular thrombus (GT) and acute tubular injury (ATI) [7]. Conversely, the Maryland aggregate pathology index (MAPI), Pirani, and Remuzzi scoring systems do not include acute lesions [1–3]. Additionally, scoring systems have different scoring criteria. The Banff scoring system employs a percentage-based approach to evaluate GS [7], while the Pirani and Remuzzi scoring systems assign ordinal grades to GS. Moreover, different scoring systems assign varying weights to histopathological lesions. Banff donor scoring system incorporates both arterial fibrosis score and arteriolar hyalinosis (AH) score, whereas the Remuzzi scoring systems only incorporates score of the most severe vascular lesion. The divergences in items and scoring criteria might result in variations in the evaluation of donor kidney quality. Currently, there is no consensus on best scoring system for assessing lesions and predict allograft prognosis. The capacities of items in predicting long-term allograft function and the reasons for different prognosis predictive capacities among systems remain unclear.

Most histopathological scoring systems for donor kidney necessitate a wedge biopsy (WB) with a minimum of 25 glomeruli [1,2]. However, WB tends to sample the subcapsular cortex with more sclerotic glomeruli, leading to GS overestimation [8,9]. Core biopsy (CB) allows for better evaluation of vascular pathology and reduces risk of GS overestimation. Most scoring systems were established based on WB. Discrepancies between WB and CB could introduce differences in adapting WB-derived scoring systems to CB [10]. The extent to which WB-derived scoring systems can predict allograft prognosis in CB remains uncertain. Furthermore, the glomerular number in CB may not meet the threshold of WB-derived scoring systems. The adequate number of glomeruli in CB was not established. Inadequate specimens might reduce the predictive capacities of scoring systems [11]. A previous study suggested that a CB with at least 10 glomeruli could adequately reflect donor kidney status [9]. Nevertheless, there is no clinical validation to confirm this proposal.

It remains unknown whether WB-derived scoring systems could predict allograft prognosis in CB and which performs best. The impact of glomerular number on the prognostic predictive capacities of scoring systems in CBs remains unclear. Furthermore, it is essential to explore strategies for enhancing the predictive capacities of scoring systems in CB.

This study aimed to explore the predictive capacity of scoring systems and items on long-term allograft function based on CB. We explored the adequate glomerular number and investigated its impact on the validity of prognostic reliability. Additionally, we developed a new modified scoring method to enhance the predictive capacity of scoring systems in CB.

## Patients and methods

### Study population

The study was a single-center, retrospective, observational cohort study. Recipients who received deceased kidney allograft at the First affiliated hospital of Sun Yat-sen University from 2014 to 2018 were enrolled. The inclusion criteria include: (1) donor ≥ 18 years old; (2) pre-transplant core biopsy in the donor allograft was performed. The exclusion criteria include: (1) En-bloc kidney transplantation; (2) combined organ transplantation.

Prior to organ procurement, we assessed donor kidney quality based on clinical profiles, including medical history and laboratory test. CB was performed on marginal donor kidneys or those with visible anomalies on kidney or renal vessels. The acceptance or discard of the kidney was determined based on a holistic evaluation of clinical profiles, kidney appearance, and pathology. Due to the long waiting list and donor scarcity, all accepted donor kidneys were for single kidney transplantation. The study was approved by the Institutional Review Board of our hospital (NO.2019-453) and conducted under the ethical principles outlined in the Declaration of Helsinki and the Declaration of Istanbul. The requirement of informed consent was waived because of its retrospective nature. All the organs were procured after informed consent or authorization for organ donation by organ procurement organization. None of the organs were procured from executed prisoners.

### Histopathological evaluation of pre-transplant kidney biopsies

The core biopsy was performed before transplantation at the upper pole of the kidney allograft using a 16-gauge core, angled at 30°–50° to the donor kidney cortical ridge. Pre-transplant histologic evaluation was conducted in frozen section. The remaining tissue would be then fixed in 4% Formaldehyde and embedded in paraffin for HE, PAS, PASM and MASSON staining later. Two experienced renal pathologists independently reviewed all the paraffin-embedded sections under light microscope, who were both blinded to the clinical data. Banff donor score, Chronic Allograft Damage Index (CADI) score, Deceased Donor Scores (DDS), MAPI score, Pirani score, Remuzzi score and Ugarte score were employed [1–7]. We additionally scored GS with three times of percentage of GS in Banff donor scoring systems. Grades of none, mild, moderate, and severe were assigned points 0 to 3, respectively. In assessing specimens with less than ten glomeruli, the GS-related score was excluded, and the item scores pertaining to chronic tubulointerstitial lesions were multiplied by a factor of 1.5. For MAPI scoring system, Scar item was duplicated. The new scoring systems were defined as the modified scoring systems. Ugarte scoring system was not modified as it lacked item scores.

A binary classification approach based on the No/Mild and moderate or severe (M-S) grading criteria was applied. It allows for practical differentiation between high and low pathological scores while ensuring sufficient sample sizes for reliable statistical analysis. Specimens with a total score of 0-3 points in Banff donor score, DDS score, Remuzzi score and Pirani score were considered as Mild, while those with a total score of 4-12 were considered as M-S. For MAPI score, specimens with a total score of 0-7 points were considered as Mild, while those with a score higher than 7 were considered M-S. For CADI score, specimens with a total score of 0-4 points were considered as Mild, while those with a score higher than 4 were considered M-S. Specimens with a total Ugarte score of 0 point were graded as No, while those with a score higher than 0 were considered M-S.

### Clinical parameters

Both donors’ and recipients’ demographic characteristics and baseline information were collected from the national and local databases. The primary outcome is estimated glomerular filtration rate (eGFR) [12], which was calculated using the Chinese modified MDRD formula and recorded as 0 if graft loss occurred. Death-censored graft failure (DCGF) was defined as graft removal, return to dialysis and re-transplantation. Delayed graft function (DGF) was defined as the required use of dialysis within one week after transplantation. All the patients received standard immunosuppressive regimens.

### Statistical analysis

Continuous data with normal distribution was presented as the mean ± standard deviation (SD), while continuous data without normal distribution was expressed as median [Q1 – Q3] and compared using Mann–Whitney U-test. The normality of the data was assessed using the Shapiro–Wilks test. Categorical data were reported as count and percentage and compared using the Chi-square test or Fisher’s exact test. Kaplan–Meier method and log-rank test were used for survival analysis. Spearman correlation was used for correlation analysis. Logistic regression and linear regression models were used for univariate and multivariate analysis of factors for allograft prognosis. Linear regression models were used to analyze the predictive capacity in eGFR of scoring systems or items. The eGFR predictive model included the total score or item score, adjusted by peri-operative variables including acute tubular injury, DGF, recipient age at transplantation, recipient body mass index (BMI), pre-transplant recipient panel reactive antibody (PRA) status, cold ischemia time and warm ischemia time. A p-value < 0.05 was considered statistically significant. The models were compared by the Vuong test. All analyses were performed using R statistical software (version 4.0.3, R Foundation for Statistical Computing, Vienna, Austria).

## Results

### Baseline characteristics

**Figure 1.**
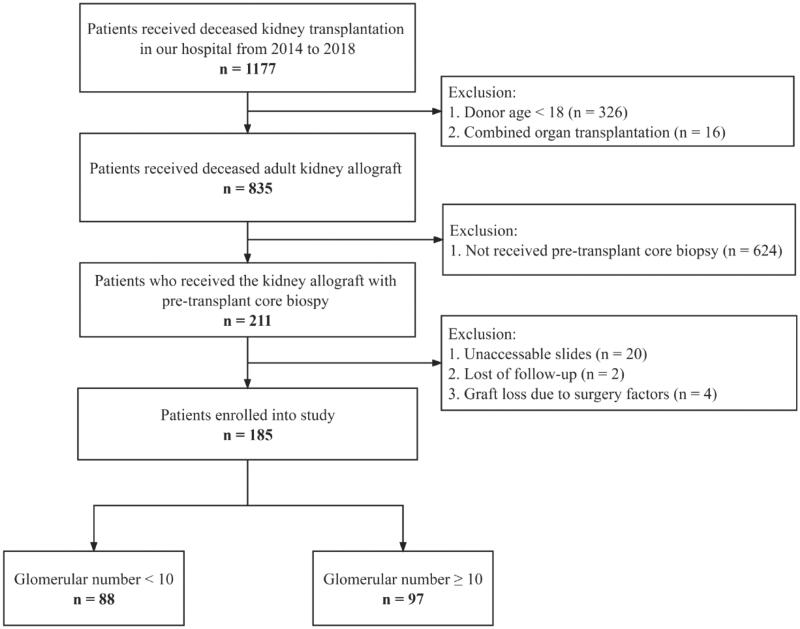
The inclusion and exclusion flowchart.

Overall, 185 recipients were enrolled (Figure 1). The specimens had a median glomerular number of 10. Specimens with a glomerular number ≥ 10 were defined as adequate specimens while others were defined as inadequate specimens. The patients were classified into two groups: a group with a glomerular number < 10 (*n* = 88, 47.6%) or a group with a glomerular number ≥ 10 (*n* = 97, 52.4%). Table 1 showed the demographic and clinical characteristics of recipients. Table S2 had shown the donor demographical and clinical profiles were comparable between enrolled allografts and allografts without PTDB.

**Table 1.** Demographic and clinical characteristics of recipients.

	Overall (*n* = 185)	Glomerular number < 10 (*n* = 88)	Glomerular number ≥ 10 (*n* = 97)
**Donor**			
Age (years)	37.78 ± 12.36	38.92 ± 13.07	36.74 ± 11.64
Weight (kg)	63.91 ± 10.61	63.03 ± 9.77	64.65 ± 11.30
Donor Type *n* (%)			
DBD	139 (75.1)	70 (79.5)	69 (71.1)
DCD	46 (24.9)	18 (20.5)	28 (28.9)
Terminal Scr (μmol/L)	102.35 [69.75, 165.40]	104.00 [70.00, 137.00]	99.00 [67.70, 192.50]
Death of cerebrovascular events n (%)	49 (26.5)	30 (34.1)	19 (19.6)
Glomerular number	10.00 [6.00, 13.00]	6.00 [4.00, 8.00]	13.00 [11.00, 18.00]
**Recipient**			
Age (years)	43.97 ± 12.80	44.20 ± 13.56	43.75 ± 12.13
Weight (kg)	59.92 ± 11.23	59.01 ± 10.35	60.75 ± 11.96
History of DM *n* (%)	26 (14.1)	14 (15.9)	12 (12.4)
PRA n (% positive)	55 (29.7)	23 (26.1)	32 (33.0)
The duration of dialysis (month)	16.28 ± 20.58	17.95 ± 20.10	14.44 ± 21.14
Re-transplantation *n* (%)	6 (3.2)	2 (2.3)	4 (4.1)
Cold ischemia time (hours)	10.19 ± 5.22	9.80 ± 5.07	10.57 ± 5.37
Warm ischemia time (minute)	2.58 ± 5.77	3.33 ± 7.36	1.89 ± 3.68
DGF n (%)	25 (13.5)	12 (13.6)	13 (13.4)
**Treatment**			
Induction n (%)			
ATG	153 (82.7)	72 (81.8)	81 (83.5)
Basiliximab	32 (17.3)	16 (18.2)	16 (16.5)
Anti-proliferative agent n (%)			
EC-MPS	122 (65.9)	59 (67.0)	63 (64.9)
Mizoribine	1 (0.5)	1 (1.1)	0 (0.0)
MMF	62 (33.5)	28 (31.8)	34 (35.1)
CNI n (%)			
Cyclosporine	4 (2.2)	2 (2.3)	2 (2.1)
Tacrolimus	181 (97.8)	86 (97.7)	95 (97.9)

DBD: donation after brain death; DCD: donation after cardiac death; HP: hypertension; DM: Diabetes mellitus; Scr: serum creatinine; PRA: panel reactive antibody; DGF: delayed graft function; ATG: anti-thymoeyte globulin; EC-MPS: enteric-coated mycophenolic sodium; MMF: mycophenolate mofetil; CNI: calcineurin inhibitor.

### Scoring systems could predict allograft prognosis based on CB with a glomerular number ≥ 10

**Figure 2.**
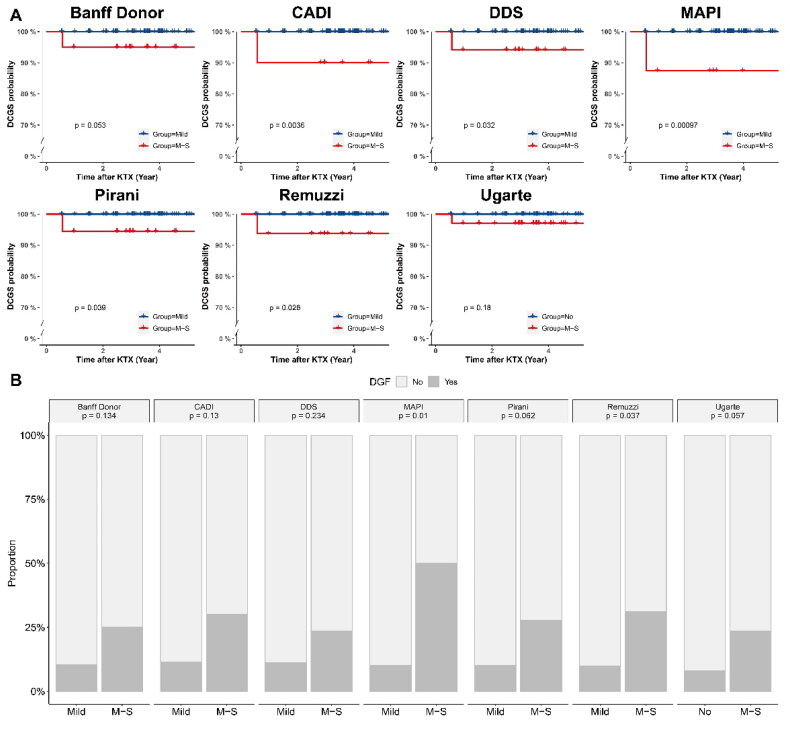
Comparison Of allograft prognosis in specimens with a glomerular number ≥ 10, stratified across each scoring system pre-defined risk groups. (A) The Kaplan-Meier death-censored graft survival curves for different scoring systems. (B) Comparison of DGF incidence between Mild/no and M-S grades.

**Figure 3.**
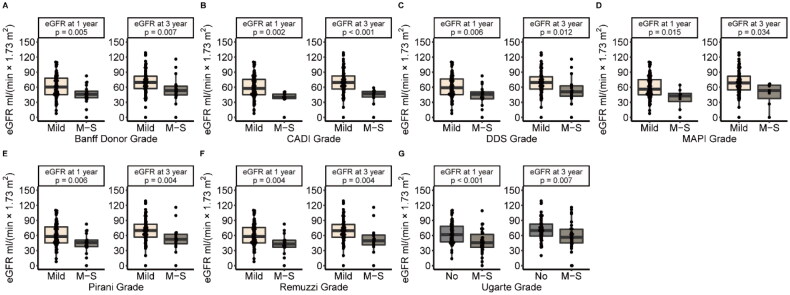
eGFR Boxplot of recipients in different grades of different scoring systems in specimens with a glomerular number ≥ 10. (A) Banff donor scoring system; (B) CADI scoring system; (C) DDS scoring system; (D) MAPI scoring system; (E) Pirani scoring system; (F) Remuzzi scoring system; (G) Ugarte scoring system.

We compared the allograft prognosis between score-based grades in different scoring systems in the group with a glomerular number ≥ 10. Figure 2(A) showed that the death-censored graft survival rate of M-S Grade was significantly lower than Mild Grade in CADI (*p* = 0.0036), DDS (*p* = 0.032), MAPI (*p* = 0.001), Pirani (*p* = 0.039) and Remuzzi (*p* = 0.026) scoring systems. Figure 2(B) showed that M-S Grade associated with higher incidence of DGF in MAPI (*p* = 0.01) and Remuzzi (*p* = 0.037) scoring systems. M-S grade of MAPI was an independent risk factor of DGF (OR = 1.59, *p* < 0.001, Table 2). eGFR at one year and three years after kidney transplantation were significantly lower in M-S Grade (*p* < 0.05) (Figure 3). Nevertheless, the M-S Grade was not significantly associated with lower eGFR in the group with a glomerular count < 10 (Figure S1).

**Table 2.** Adjusted* odd ratios (or) for DGF in recipients receiving allografts with CB specimens’ glomerular number ≥ 10.

Scoring system**	OR	95%CI	*P* value
Banff donor	0.99	0.85 − 1.15	0.869
CADI	1.10	0.84 − 1.42	0.485
DDS	1.08	0.86 − 1.37	0.503
MAPI	1.59	1.24 − 2.04	**<0.001**
Pirani	1.14	0.93 − 1.39	0.198
Remuzzi	1.2	0.96 − 1.50	0.101
Ugarte	1.12	0.96 − 1.30	0.165

* Adjusted by acute tubular injury grade (Mild vs. No Grade), cold ischemia time and donor creatinine.

** M-S Grade vs. Mild/No Grade.

The bold value was for the emphasis of significant *p* value.

**Figure 4.**
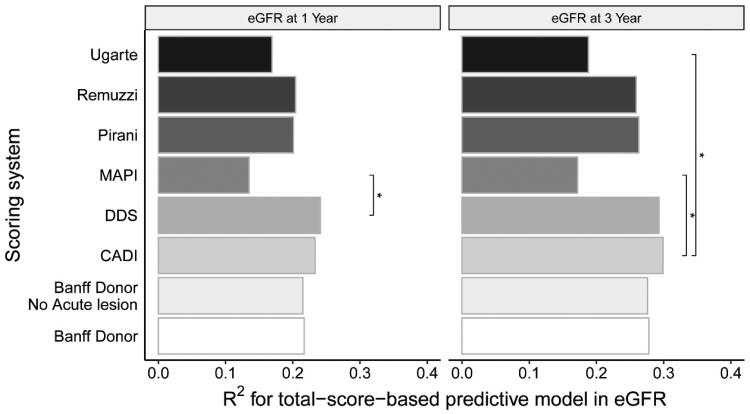
Comparison Of the goodness of fit of linear regression models established in specimens with a glomerular number ≥ 10. The total-score-based models predicted eGFR. The goodness of fit of each model was compared with the DDS scoring system for one-year eGFR prediction and CADI for 3-year eGFR prediction, by the Vuong test. Label * would be assigned only if a model differed significantly from the reference model.

All scoring systems were significantly associated with a lower eGFR. The DDS score best explained lower one-year eGFR (R^2^_max_ = 0.241, *p* < 0.001) and CADI score best explained three-year eGFR (R^2^_max_ = 0.299, *p* < 0.001) (Figure 4) in the group with a glomerular number ≥ 10. The DDS and CADI scoring system were the reference scoring system because of the highest predictive capacity. Only the MAPI and Ugarte scoring system were significantly different from reference model (*p* < 0.05) (Figure 4).

Figure S2 showed the goodness of fit of linear regression models that predict eGFR based on different item scores. The GS, IF, TA and AH had generally higher predictive capacities than other items across different scoring systems. The GT and ATI had significantly lower predictive capacities than other chronic lesions. Among the Banff donor, Remuzzi, DDS and Pirani scoring systems, GS scores correlated with each other and had similar predictive capacities in eGFR (*p* > 0.05) (Figure S3).

### The application of modified scoring systems in specimens with a glomerular number < 10 could improve predictive capacity in eGFR

**Figure 5.**
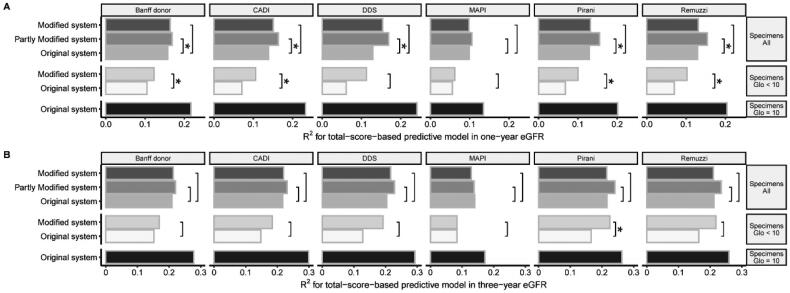
Comparison Of the goodness of fit of linear regression models. (A) One-year eGFR prediction; (B) Three-year eGFR prediction. The total-score-based models predicted eGFR in different specimens. The goodness of fit of each model was compared with the original scoring system as the reference scoring system, by the Vuong test. Label * would be assigned only if a model differed significantly from the reference model.

When assessing inadequate specimens, the scoring system was modified. After modification, the grading of scoring systems, except for MAPI and CADI, was associated with post-transplant eGFR (Figure S4). The modified Banff donor, CADI, Pirani and Remuzzi scoring systems could better predict one-year eGFR than the original system (*p* < 0.05) (Figure 5). Modified scoring systems had higher R^2^ values in predicting three-year eGFR, except for MAPI. The modified Pirani scoring systems could better predict three-year eGFR than the original systems (*p* < 0.05) (Figure 5).

We compared the predictive capacities of models in adequate or inadequate specimens. The models in adequate specimens had the highest goodness of fit (Figure 5). We built the partly modified scoring systems by applying modified system only in specimens with a glomerular number < 10. The partly modified scoring systems generally had a higher predictive capacity than the original scoring system, except for the MAPI system (Figure 5).

## Discussion

Our study demonstrated that all scoring systems could stratify risk with a lower eGFR in CB with more than 10 glomeruli. The eGFR predictive capacities of scoring systems were similar, except for the Ugarte and MAPI scoring systems. GS, IF, TA, and AH items were major contributors to the eGFR prediction across different scoring systems (Figure S3), which may explain the comparable predictive capacities of scoring systems. Furthermore, removing inadequate GS scores while adjusting the weights of chronic tubulointerstitial scores improved the predictive capacity in CB specimens.

DDS and CADI exhibited superior predictive capacities in predicting post-transplant eGFR, while MAPI and Ugarte were significantly inferior to DDS and CADI. The DDS and CADI systems scored chronic lesions with three-level criteria and used total score to indicate allograft quality. In contrast, MAPI employed binary classification approach, while the Ugarte system employed grades without scores. These findings suggested that the disparities among scoring systems might result in difference in eGFR predicting capacities. Not all pathological scoring systems work well in predicting long-term eGFR. Therefore, it was crucial to compare the predictive capacities of scoring systems and select the best one for long-term post-transplant eGFR prediction.

As of yet, no consistent evidence exists that all items can contribute to the prediction of long-term allograft function. C. J. Wang et al. reported substantial heterogeneity in the prognosis predictive capacity of different scoring systems and items across different studies [11], with some leading to contradictory conclusions. Nevertheless, Wang’s study did not validate and compare the prognosis predictive capacities of items within the same cohort. Additionally, Stewart et al. investigated and compared the predictive capacities of different items [13]. GS was found to be an independent risk factor for allograft survival. Vascular and interstitial lesions was not found be risk factors. Nevertheless, the prognostic predictive capacities on long-term eGFR of different pathological lesions in PTDB remain unknown. Currently, it was unclear which chronic lesion in PTDB had a greater predictive capacity for long-term eGFR. It was crucial for the establishment of a simple but practical PTDB scoring system for long-term allograft prognosis prediction.

The predictive capacities of GS scores varied across different scoring systems, possibly attributed to differing scoring criteria. Banff donor scoring system assessed the proportion of GS quantitatively, while the threshold for severe GS ranged from 15% to 50% among other systems. GS score in Banff donor scoring system had comparable correlation and predictive capacity with other scoring systems using ordinal grading criteria, such as DDS, Pirani and Remuzzi scoring systems. MAPI’s dichotomous scoring criterion on GS had no further subdivision of GS exceeding 15%, result in possible misestimation of GS severity. Nevertheless, GS score of MAPI scoring system had similar predictive capacity with that of Remuzzi system in our study. A possible explanation is that the about 70.3% of the adequate specimens share identical GS score for both MAPI and Remuzzi systems. The predictive capacities of the two scoring systems were similar as their scores were closely aligned. In overall eGFR prediction, Remuzzi and Pirani scoring systems demonstrated stable and effective predictive capabilities, while MAPI performed worse in predicting one-year eGFR. We proposed that three-level grading criteria of Remuzzi and Pirani scoring system might better reflect the impact of GS on long-term eGFR. The comparison between the dichotomous and three-level grading criteria necessitates further research. Regarding pathological vascular changes, it remained unclear whether AH or vascular fibrous intimal thickening (CV) items could better predict allograft prognosis. AH is associated with deteriorating renal function and found to be a risk factor for advancing GS [14,15]. In clinical observation, we noticed that a high AH score was associated with poor prognosis, even in donor kidney with a low total score. In our study, AH showed a higher predictive capacity for eGFR than CV. We proposed that AH was more important than CV in predicting allograft prognosis.

According to our result, scoring systems had similar predictive capacities for eGFR. GS had dominant predictive capacity on eGFR and was used as a standard to compare the predictive capacities of items. GS, IF, TA and AH items had satisfactory but different predictive capacities in long-term eGFR. Interstitial inflammation was a risk factor for allograft prognosis and had a comparable predictive capacity for eGFR [16]. Conversely, acute lesions such as ATI and GT displayed weaker eGFR predictive capacities. The inclusion of acute lesion scores into the total score did not improve predictive capacity in eGFR (Figure S2). It was consistent with previous studies that acute lesions did not affect long-term eGFR [17,18]. Furthermore, PGF and wall-lumen ratio (WLR) had weaker predictive abilities, compared to common chronic lesion.

In summary, we suggested that the GS, IF, TA and AH were the satisfactory predictors of post-transplant eGFR. A scoring system that incorporates these chronic lesions could be simple and pragmatic in kidney evaluation with good long-term eGFR predictive capacity [19]. Wang et al. raised that ATI in donor kidney CB could be employed to predict DGF^18^. For these reasons, we proposed that a pragmatic scoring system should include both acute and chronic lesions to predict DGF and long-term prognosis. The new scoring system should weight items differently, considering their varying predictive capacities. Notably, only MAPI scoring system and its PGF item could predict DGF. The predictive capacity of the PGF item in DGF should be validated in further research.

Scoring systems were not able to stratify recipients with risk of lower post-transplant eGFR in inadequate specimens. It might be due to GS misestimation. It was observed that in CB with fewer than 10 glomeruli, the severity of GS was incorrectly assessed in 69% cases [9]. The GS misestimation resulted in poor predictive capacity. Our findings support the proposition that a glomerular threshold of 10 could be deemed sufficient for evaluating donor kidney CBs.

When biopsy was inadequate, we had to accept or discard the donor kidneys based on clinical profiles in the absence of GS data. Pathologists usually need a second biopsy specimens for evaluation. Despite, performing a second kidney biopsy increases the risk of postoperative hemorrhage and urinary fistula. Hence, we attempted to reevaluate inadequate CB specimens without second biopsy. Modified scoring systems, except CADI and MAPI, could distinguish recipients with lower eGFR. Previous studies proposed that GS was associated with consequent TA and IF [20–25], which were crucial predictors of long-term renal function [21,26–28]. Mazzucco et al. reported that the agreement between actual donor kidney status and CB specimen with a glomerular number < 10 was acceptable for IF and TA^9^. Thus, we proposed that the IF and TA scores could be feasible indicator of allograft lesions even with inadequate GS assessment. Hence, we removed inaccurate GS scores and adjusted the weights of IF and TA scores, aligning the total score of the modified scoring system with the original one. This allows for the direct application of the clinical grading criteria from the original system, facilitating a more convenient assessment in clinical practice.

To the best of our knowledge, it is the first study to remove GS score and adjust the weights of IF and TA scores in inadequate CB specimen evaluation. It provides additional prognostic value to the overall assessment of donor kidney. The modified scoring systems had improved predictive capacity for eGFR in inadequate specimens. The most effective scheme for donor kidney evaluation was to apply the original scoring system to adequate CB specimens and the modified scoring systems to inadequate CB specimens. The further predictive capacity of modified scoring system in CB specimens need to be validated in larger sample size.

In our study, most scoring systems could stratify the risk of DCGF excluding the Ugarte system, which was consistent with previous study [5]. The MAPI scoring system had a significant predictive capacity on DGF (OR = 1.59, *p* < 0.001). Only PGF item was an independent risk factor of DGF (OR = 1.06, *p* = 0.006).

Our study had several limitations. First, potential selection bias might exist in the decision to perform a PTDB. Nevertheless, PTDB cohort had similar clinical profiles to donor kidneys without PTDB (Table S2), reducing selection bias and rendering it more representative of the overall donor kidney population instead of expanded criteria donor kidneys. It helps enhance the applicability of study results to overall donor kidneys. Secondly, we used consecutive numbers ranging from 0 to 3 to replace qualitative ordinal grades in the scoring systems. However, assigning scores based on the relative impact of each ordinal grade on the prognosis might provide more accurate results. Therefore, future studies could consider using a more nuanced scoring system to improve the precision of the scoring system. This study was based on paraffin sections, and the feasibility of its findings on frozen sections requires further investigation.

In conclusion, CB is important for assessing donor kidney quality. Our study found that most scoring systems had similar predictive capacities in eGFR for donor kidney CB specimens with more than 10 glomeruli, except for the MAPI and Ugarte scoring system. We recommend the development of a pragmatic scoring system that incorporates acute and chronic lesions, such as GS, IF, TA, and AH, to predict short- and long-term prognosis. It is important to assign appropriate weights to each item based on their relative contribution to prognosis prediction. Furthermore, chronic tubulointerstitial injury should be emphasized in inadequate specimens to predict post-transplant prognosis. The conclusion should be further validated in frozen sections.

## Supplementary Material

Supplemental Material

## References

[1] Remuzzi G, Grinyò J, Ruggenenti P, et al. Early experience with dual kidney transplantation in adults using expanded donor criteria. Double kidney transplant group (DKG). J Am Soc Nephrol. 1999;10(12):1–9. Dec doi: 10.1681/ASN.V10122591. 10589699

[2] Karpinski J, Lajoie G, Cattran D, et al. Outcome of kidney transplantation from high-risk donors is determined by both structure and function. Transplantation. 1999;67(8):1162–1167. doi: 10.1097/00007890-199904270-00013. 10232568

[3] Munivenkatappa RB, Schweitzer EJ, Papadimitriou JC, et al. The Maryland aggregate pathology index: a deceased donor kidney biopsy scoring system for predicting graft failure. Am J Transplant. 2008;8(11):2316–2324. doi: 10.1111/j.1600-6143.2008.02370.x. 18801024

[4] Isoniemi H, Taskinen E, Häyry P. Histological chronic allograft damage index accurately predicts chronic renal allograft rejection. Transplantation. 1994;58(11):1195–1198. 7992362

[5] Ugarte R, Kraus E, Montgomery RA, et al. Excellent outcomes after transplantation of deceased donor kidneys with high terminal creatinine and mild pathologic lesions. Transplantation. 2005;80(6):794–800. doi: 10.1097/01.tp.0000173801.33878.bf. 16210967

[6] Azancot MA, Moreso F, Salcedo M, et al. The reproducibility and predictive value on outcome of renal biopsies from expanded criteria donors. Kidney Int. 2014;85(5):1161–1168. doi: 10.1038/ki.2013.461. 24284518

[7] Liapis H, Gaut JP, Klein C, et al. Banff histopathological consensus criteria for preimplantation kidney biopsies. Am J Transplant. 2017;17(1):140–150. doi: 10.1111/ajt.13929. 27333454 PMC6139430

[8] Carpenter D, Husain SA, Brennan C, et al. Procurement biopsies in the evaluation of deceased donor kidneys. Clin J Am Soc Nephrol. 2018;13(12):1876–1885. doi: 10.2215/CJN.04150418. 30361336 PMC6302333

[9] Mazzucco G, Magnani C, Fortunato M, et al. The reliability of pre-transplant donor renal biopsies (PTDB) in predicting the kidney state. A comparative single-Centre study on 154 untransplanted kidneys. Nephrol Dial Transplant. 2010;25(10):3401–3408. doi: 10.1093/ndt/gfq166. 20356979

[10] Yong ZZ, Kipgen D, Aitken EL, et al. Wedge versus core biopsy at time zero: which provides better predictive value for delayed graft function with the remuzzi histological scoring system? Transplant Proc. 2015;47(6):1605–1609. doi: 10.1016/j.transproceed.2015.03.050. 26293021

[11] Wang CJ, Wetmore JB, Crary GS, et al. The donor kidney biopsy and its implications in predicting graft outcomes: a systematic review. Am J Transplant. 2015;15(7):1903–1914. doi: 10.1111/ajt.13213. 25772854

[12] Ma YC, Zuo L, Chen JH, et al. Modified glomerular filtration rate estimating equation for Chinese patients with chronic kidney disease. J Am Soc Nephrol. 2006;17(10):2937–2944. doi: 10.1681/ASN.2006040368. 16988059

[13] Stewart DE, Foutz J, Kamal L, et al. The independent effects of procurement biopsy findings on 10-year outcomes of extended criteria donor kidney transplants. Kidney Int Rep. 2022;7(8):1850–1865. doi: 10.1016/j.ekir.2022.05.027. 35967103 PMC9366372

[14] Moriya T, Omura K, Matsubara M, et al. Arteriolar hyalinosis predicts increase in albuminuria and GFR decline in normo- and microalbuminuric Japanese patients with type 2 diabetes. Diabetes Care. 2017;40(10):1373–1378. doi: 10.2337/dc17-0209. 28774945

[15] Bazzi C, Stivali G, Rachele G, et al. Arteriolar hyalinosis and arterial hypertension as possible surrogate markers of reduced interstitial blood flow and hypoxia in glomerulonephritis. Nephrology (Carlton). 2015;20(1):11–17. doi: 10.1111/nep.12339. 25230383

[16] Raynaud M, Aubert O, Reese PP, et al. Trajectories of glomerular filtration rate and progression to end stage kidney disease after kidney transplantation. Kidney Int. 2021;99(1):186–197. doi: 10.1016/j.kint.2020.07.025. 32781106

[17] Batra RK, Heilman RL, Smith ML, et al. Rapid resolution of donor-derived glomerular fibrin thrombi after deceased donor kidney transplantation. Am J Transplant. 2016;16(3):1015–1020. doi: 10.1111/ajt.13561. 26689853

[18] Wang J, Liu J, Wu W, et al. Combining clinical parameters and acute tubular injury grading is superior in predicting the prognosis of deceased-donor kidney transplantation: a 7-year observational study. Front Immunol. 2022;13:912749. doi: 10.3389/fimmu.2022.912749. 35844570 PMC9279653

[19] Sethi S, D'Agati VD, Nast CC, et al. A proposal for standardized grading of chronic changes in native kidney biopsy specimens. Kidney Int. 2017;91(4):787–789. doi: 10.1016/j.kint.2017.01.002. 28314581

[20] Roberts ISD, Cook HT, Troyanov S, Working Group of the International Ig ANN, the Renal Pathology S., et al. The oxford classification of IgA nephropathy: pathology definitions, correlations, and reproducibility. Kidney Int. 2009;76(5):546–556. doi: 10.1038/ki.2009.168. 19571790

[21] Yu F, Wu LH, Tan Y, et al. Tubulointerstitial lesions of patients with lupus nephritis classified by the 2003 international society of nephrology and renal pathology society system. Kidney Int. 2010;77(9):820–829. doi: 10.1038/ki.2010.13. 20182417

[22] Kriz W, LeHir M. Pathways to nephron loss starting from glomerular diseases-insights from animal models. Kidney Int. 2005;67(2):404–419. doi: 10.1111/j.1523-1755.2005.67097.x. 15673288

[23] Fine LG, Norman JT. Chronic hypoxia as a mechanism of progression of chronic kidney diseases: from hypothesis to novel therapeutics. Kidney Int. 2008;74(7):867–872. doi: 10.1038/ki.2008.350. 18633339

[24] Schlondorff DO. Overview of factors contributing to the pathophysiology of progressive renal disease. Kidney Int. 2008;74(7):860–866. doi: 10.1038/ki.2008.351. 18650795

[25] Remuzzi G. A unifying hypothesis for renal scarring linking protein trafficking to the different mediators of injury. Nephrol Dial Transplant. 2000;15(suppl_6):58–60. doi: 10.1093/ndt/15.suppl_6.58. 11143993

[26] Moura MC, Fervenza FC, Specks U, et al. Kidney biopsy chronicity grading in antineutrophil cytoplasmic antibody associated vasculitis. Nephrol Dial Transplant. 2021;37(9):1710–1721. doi: 10.1093/ndt/gfab250. PMC9395375 34436585

[27] Ellingsen AR, Jørgensen KA, Østerby R, et al. Human kidney graft survival correlates with structural parameters in baseline biopsies: a quantitative observational cohort study with more than 14 years’ follow-up. Virchows Arch. 2021;478(4):659–668. doi: 10.1007/s00428-020-02924-3. 32986179

[28] Coppo R, D'Arrigo G, Tripepi G, et al. Is there long-term value of pathology scoring in immunoglobulin a nephropathy? A validation study of the oxford classification for IgA nephropathy (VALIGA) update. Nephrol Dial Transplant. 2020;35(6):1002–1009. doi: 10.1093/ndt/gfy302. 30418652

