# Supplementary material for: Value of original and modified pathological scoring systems for prognostic prediction in paraffin-embedded donor kidney core biopsy
Source: Ren Fail. 2024 Feb 12;46(1):2314630. doi: 10.1080/0886022X.2024.2314630 (PMC10863519; doi:10.1080/0886022X.2024.2314630)
Supplement: Supplemental Material [file IRNF_A_2314630_SM1447.pdf]

Supplementary Material

Supplementary Tables

Table S1 Scoring criteria of different scoring systems

Table S2 Comparison of the donor demographical and clinical characteristics between allografts enrolled and allografts without PTDB.

Supplementary Figures

Figure S1 eGFR boxplot of recipients in different grades of different scoring systems in specimens with a glomerular number <10. (A) Banff donor scoring system; (B) CADI scoring system; (C) DDS scoring system; (D) MAPI scoring system; (E) Pirani scoring system; (F) Remuzzi scoring system; (G) Ugarte scoring system.

Figure S2: Comparison of the goodness of fit of linear regression models established in specimens with a glomerular number ≥ 10. (A) One-year eGFR prediction; (B) Three-year eGFR prediction. The item-score-based models predicted eGFR. Score of scar and score of mesangial matrix increase were 0 so that the linear regression models could not be established. The goodness of fit of each model was compared with that of GS score as the reference model, by the Vuong test. Label * would be assigned only if a model differed significantly from the reference model. GS: glomerulosclerosis; IF: interstitial fibrosis; TA: tubular atrophy; CV: chronic vascular change; AH: arteriolar hyaline thickening; TI: total interstitial inflammation; GT: glomerular thrombus; ATI: acute tubular injury; I: interstitial inflammation; PGF: peri-glomerular fibrosis; WLR: wall-lumen ratio.

Figure S3: Comparison of GS score in different scoring systems. (A) Correlation heatmap; (B) Comparison of the goodness of fit of GS-score-based linear regression models in predicting eGFR. The goodness of fit of each model was compared with that of Banff donor scoring system as reference by the Vuong test. Label * would be assigned only if a model differed significantly from the reference model. GS: glomerulosclerosis.

Figure S4 Comparison of allograft prognosis in specimens with a glomerular number ＜ 10, stratified across each modified scoring system pre-defined risk groups. (A) Banff donor scoring system; (B) CADI scoring system; (C) DDS scoring system; (D) MAPI scoring system; (E) Pirani scoring system; (F) Remuzzi scoring system

Table S1 Scoring criteria of different scoring systems

Scoring systems	Score of GS 0	Score of GS 1	Score of GS 2	Score of GS 3	Score of IF 0	Score of IF 1	Score of IF 2	Score of IF 3	Score of TA 0	Score of TA 1	Score of TA 2	Score of TA 3
Banff Donor		3 × Percentage of GS			< 5%	6 – 25%	26 – 50%	> 50%	0	< 25%	26 – 50%	> 50%
CADI	0	1 - 15%	16 - 50%	> 50%	< 5%	6 – 25%	26 – 50%	> 50%	0	1 – 25%	26 – 50%	> 50%
DDS	0	1 - 10%	11 - 20%	> 20%	< 5%	6 – 25%	26 – 50%	> 50%	0	1 – 25%	26 – 50%	> 50%
MAPI	0	-	≥ 15%	-				0: Absent<br>3: Present				
Pirani	0	1 - 20%	21 - 50%	> 50%	0	< 20%	21 – 50%	> 50%	0	< 20%	21 – 50%	> 50%
Remuzzi	0	1 - 20%	21 - 50%	> 50%	0	< 20%	21 – 50%	> 50%	0	< 20%	21 – 50%	> 50%
Ugarte	0: None;<br>1: Abnormal but none of the following abnormalities;<br>2: ≥ 10%GS, Moderate-Severe fibrosis, Tubular Atrophy, Hypertensive arteriolopathy, hyaline change											

GS: glomerulosclerosis; IF: interstitial fibrosis; TA: tubular atrophy

Table S1 Scoring criteria of different scoring systems (Continued)

Scoring systems	Score of AH 0	1	2	3	Score of CV* 0	1	2	3	Specific subitem scores Subitem	0	1	2	3
Banff Donor	0	1	> 1	Multiple	0	< 25%	26 - 50%	> 50%	Interstitial inflammation	< 10%	10 - 25%	26 - 50%	> 50%
									Glomerular thrombi	None**	Mild**	Moderate**	Severe**
									Acute tubular injury	None†	Mild†	Moderate†	Severe†
CADI	-				0	< 25%	26 - 50%	> 50%	Interstitial inflammation	< 10%	10 - 25%	26 - 50%	> 50%
									Mesangial matrix increase	0	< 25%	26 - 50%	> 50%
DDS	0	1	> 1	Multiple	0	< 25%	26 - 50%	> 50%	-				
MAPI	0: Absent<br>4: Present				0: Absent<br>2: WLR ≥ 0.5				PGF	0: Absent;<br>4: Present			
Pirani	Absent	Wall thickness < Diameter of lumen	Wall thickness ≥ Diameter of lumen	Extreme	Absent	Wall thickness < Diameter of lumen	Wall thickness ≥ Diameter of lumen	Extreme	-				
Remuzzi	Absent	Wall thickness < Diameter of	Wall thickness ≥ Diameter of	Extreme	Absent	Wall thickness < Diameter of	Wall thickness ≥ Diameter of	Extreme	-				

	lumen	lumen	lumen	lumen
Ugarte	0: None;<br>1: Abnormal but none of the following abnormalities;<br>2: ≥ 10%GS, Moderate-Severe fibrosis, Tubular Atrophy, Hypertensive arteriolopathy, hyaline change			

AH: arteriolar hyaline thickening; CV: chronic vascular change (Arterial intimal fibrosis); WLR: wall-lumen ratio; PGF: peri-glomerular fibrosis

* Scored by percentage of narrowing of vascular lumen

** Mild: <10% of capillaries occluded; Moderate: 10-25% occlusion; Severe: >25% occlusion

† Mild: epithelial fattening, tubule dilation, nuclear dropout, loss of brush border; Moderate: focal coagulative type necrosis; Severe: infarction.

Table S2 Comparison of the demographical and clinical characteristics between allografts enrolled and without PTDB.

	Overall (n = 809)	Enrolled Allografts (n = 185)	Allografts without PTDB (n = 624)	P Value
**Donor**				
Age (years)	37.80 ± 11.87	37.78 ± 12.36	37.81 ± 11.74	0.977
Weight (kg)	64.07 ± 10.58	63.91 ± 10.61	64.10 ± 10.59	0.863
Donor Type (%)				0.619
DBD	621 (76.8)	139 (75.1)	482 (77.2)	
DCD	188 (23.2)	46 (24.9)	142 (22.8)	
Terminal Scr (μmol/L)	89.00 [63.00, 146.00]	102.35 [69.75, 165.40]	88.00 [62.00, 143.10]	0.064
Death of cerebrovascular events (%)	210 (26.0)	49 (26.5)	161 (25.8)	0.927
Cold ischemia time (hours)	9.96 ± 5.12	10.19 ± 5.22	9.90 ± 5.09	0.518
Warm ischemia time (minute)	3.14 ± 5.74	2.58 ± 5.77	3.29 ± 5.72	0.162

PTDB: pre-transplant donor kidney biopsy

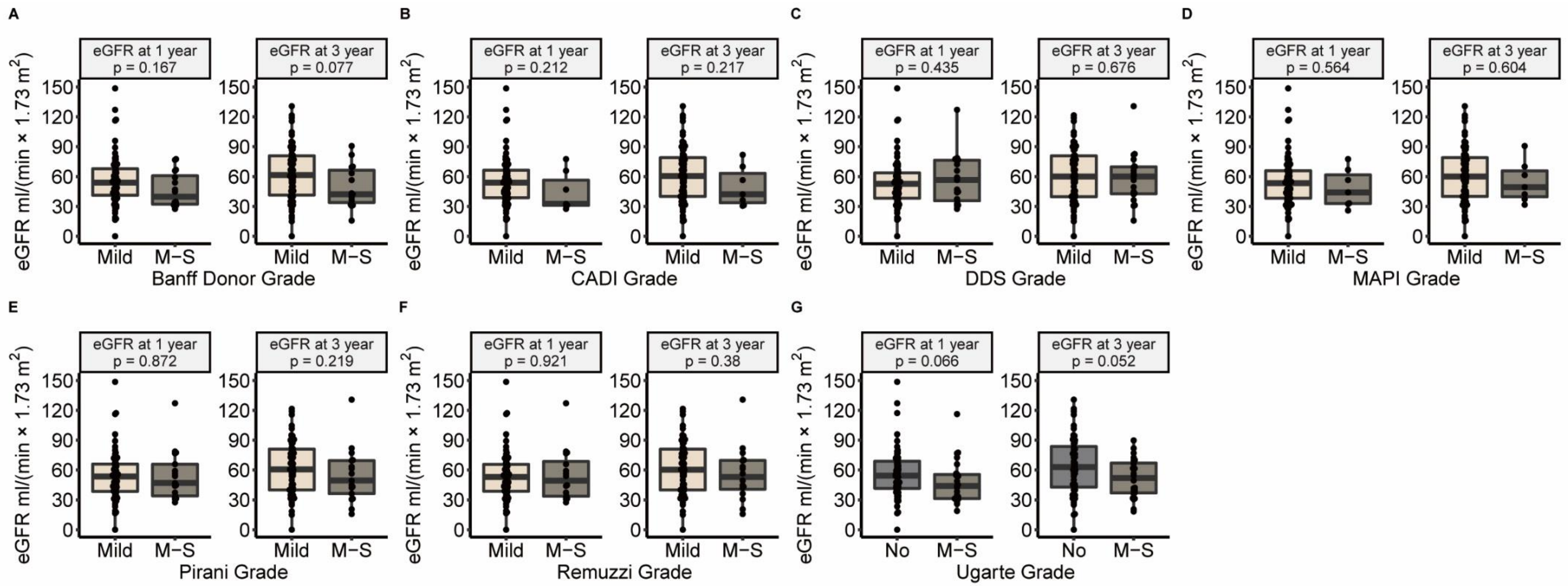

Figure S1 eGFR boxplot of recipients in different grades of different scoring systems in specimens with a glomerular number <10. (A) Banff donor scoring system; (B) CADI scoring system; (C) DDS scoring system; (D) MAPI scoring system; (E) Pirani scoring system; (F) Remuzzi scoring system; (G) Ugarte scoring system.

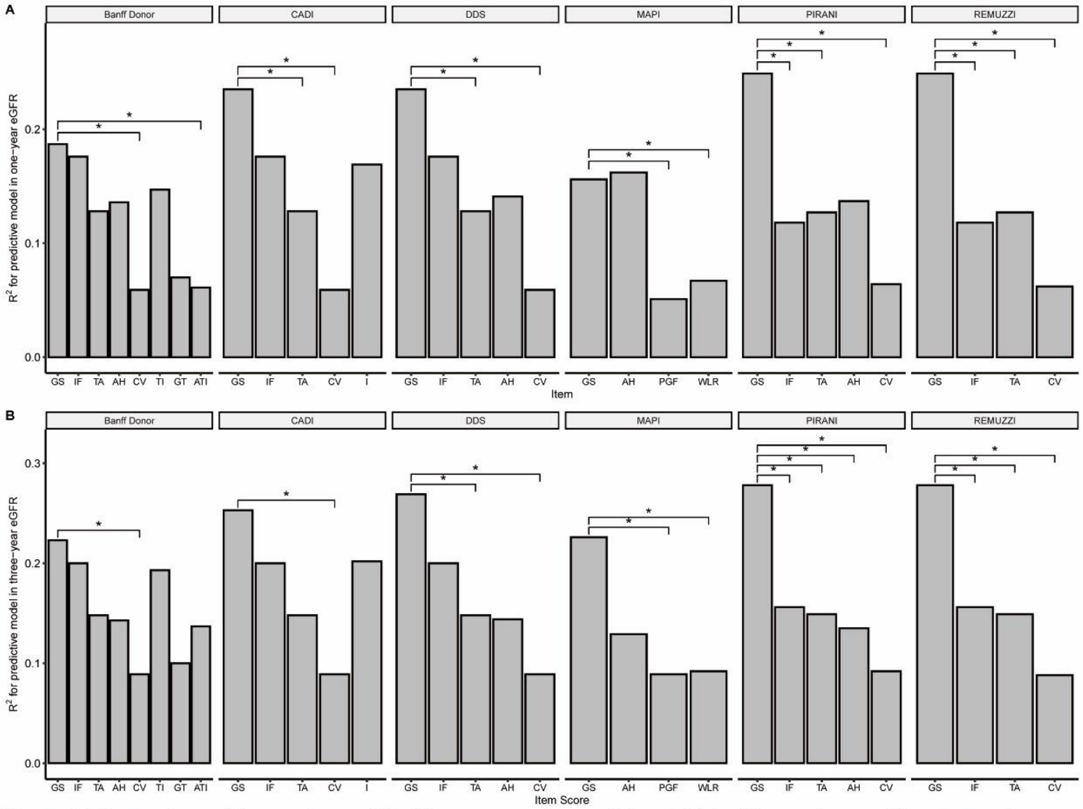

Figure S2 Comparison of the goodness of fit of linear regression models established in specimens with a glomerular number ≥ 10. (A) One-year eGFR prediction; (B) Three-year eGFR prediction. The item-score-based models predicted eGFR. Score of scar and score of mesangial matrix increase were 0 so that the linear regression models could not be established. The goodness of fit of each model was compared with that of GS score as the reference model, by the Vuong test. Label * would be assigned only if a model differed significantly from the reference model. GS: glomerulosclerosis; IF: interstitial fibrosis; TA: tubular atrophy; CV: chronic vascular change; AH: arteriolar hyaline thickening; TI: total interstitial inflammation; GT: glomerular thrombus; ATI: acute tubular injury; I: interstitial inflammation; PGF: peri-glomerular fibrosis; WLR: wall-lumen ratio

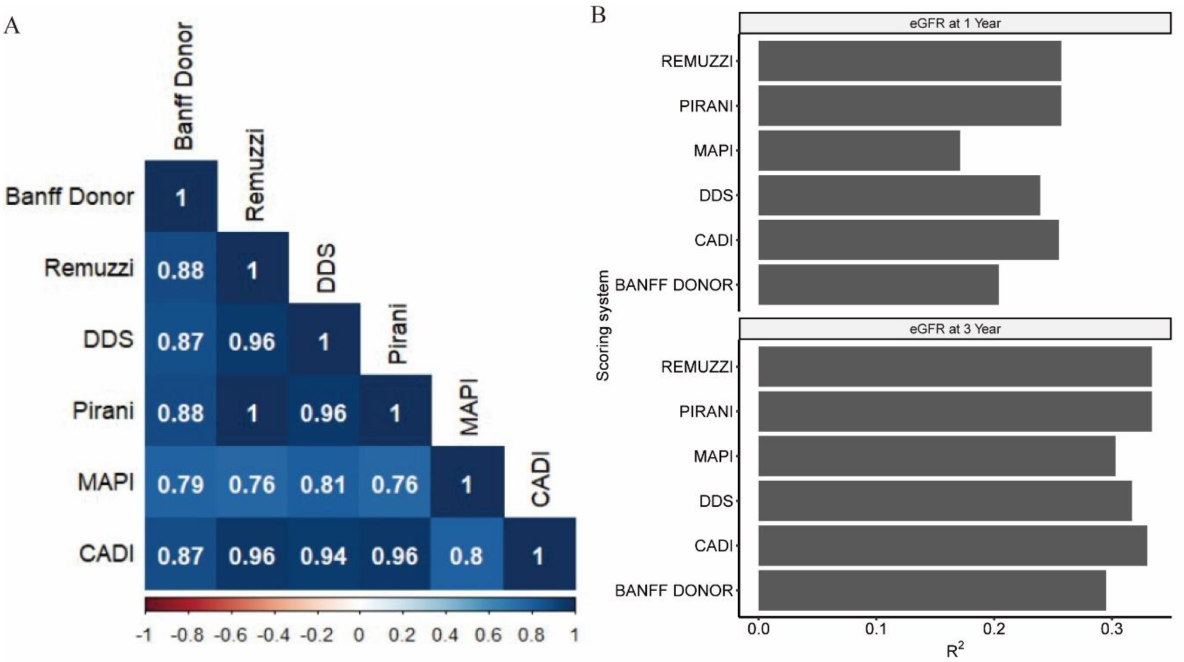

Figure S3 Comparison of GS score in different scoring systems. (A) Correlation heatmap; (B) Comparison of the goodness of fit of GS-score-based linear regression models in predicting eGFR. The goodness of fit of each model was compared with that of Banff donor scoring system as reference by the Vuong test. Label * would be assigned only if a model differed significantly from the reference model. GS: glomerulosclerosis.

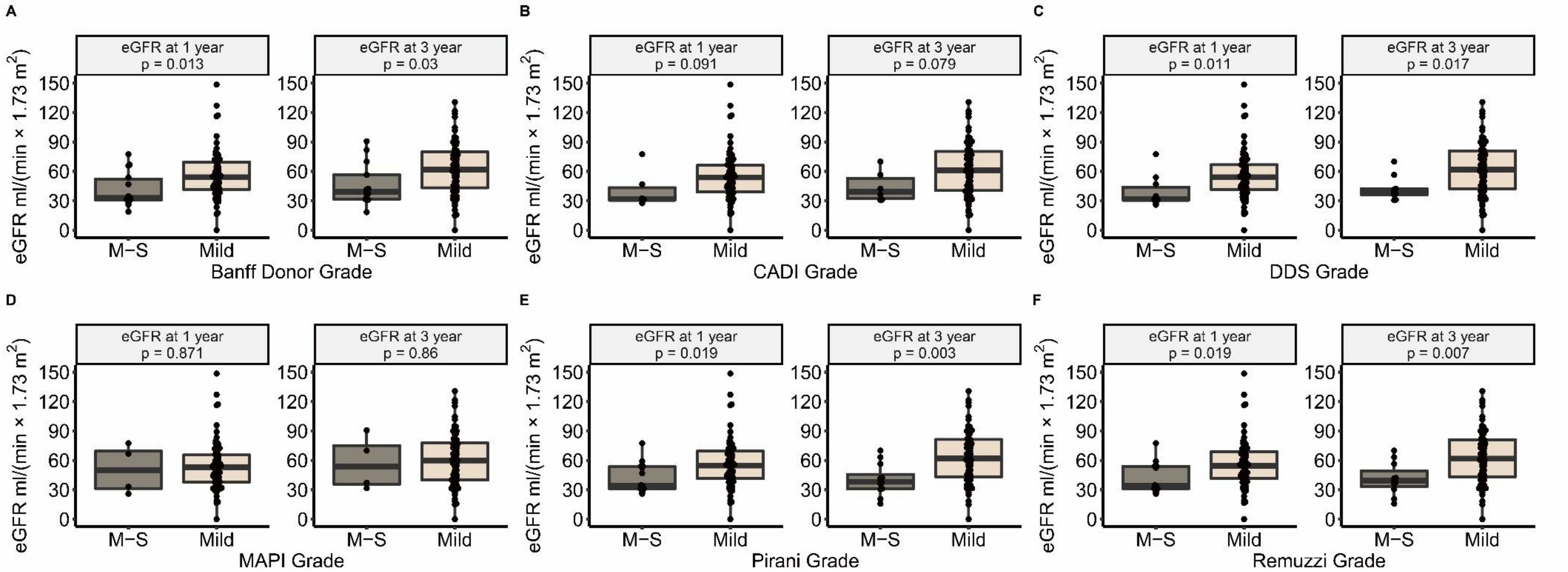

Figure S4 Comparison of allograft prognosis in specimens with a glomerular number $<$ 10, stratified across each modified scoring system pre-defined risk groups. (A) Banff donor scoring system; (B) CADI scoring system; (C) DDS scoring system; (D) MAPI scoring system; (E) Pirani scoring system; (F) Remuzzi scoring system
